# Characteristics of keratoconic patients at two main eye centres in Palestine: a cross-sectional study

**DOI:** 10.1186/s12886-018-0762-x

**Published:** 2018-04-16

**Authors:** Yousef Shanti, Ithar Beshtawi, Sa’ed H. Zyoud, Ahlam Abu-Samra, Areen Abu-Qamar, Reem Barakat, Reham Shehada

**Affiliations:** 1 Present Address: Department of Ophthalmology, An-Najah National University Hospital, 44839 Nablus, Palestine Palestine; 2 Department of Medicine, College of Medicine and Health Sciences, An-Najah National University, 44839 Nablus, Palestine Palestine; 3 Department of Optometry, College of Medicine and Health Sciences, An-Najah National University, 44839 Nablus, Palestine Palestine; 4 Department of Clinical and Community Pharmacy, College of Medicine and Health Sciences, An-Najah National University, 44839 Nablus, Palestine Palestine

**Keywords:** Keratoconus, Demographic, Palestine

## Abstract

**Background:**

Keratoconus (KC) is a multifactorial, degenerative ectatic condition of the cornea. It usually manifests during late adolescence or the early twenties. A painless disease, KC may end with severe visual loss. The prevalence of KC in middle-eastern countries is much higher than in other regions of the world. This may be due to genetic and environmental risk factors and consanguinity. The goal of this study is to explore the demographic profile of Palestinian keratoconic patients.

**Methods:**

A retrospective study was conducted in two ophthalmology centres (Tertiary Ophthalmic Centre of An-Najah National University Hospital and An-Noor Centre at the Specialized Arab Hospital). All medical charts of keratoconic patients attending both centres over the period from 2009 to 2016 were reviewed. These patients were diagnosed by ophthalmologists depending on history, examination and Pentacam. Severity was determined using the k median index from the Pentacam map. Data analysis was carried out using SPSS Version 22.

**Results:**

The medical files of 936 keratoconic eyes of 505 keratoconic patients were reviewed. Their mean age at the time of diagnosis was 23.3 ranging from 8 to 62 years. Approximately 70.1% of them presented after the age of 20 years, and younger age groups were more likely to develop a severe disease stage than older ones (*P* = 0.001, *r* = − 0.108). There was a nearly equal distribution of patients between the two sexes (49.5% male, 50.5% female). On initial evaluation, the best-corrected visual acuity (BCVA) was recorded as ≥6/12 in most affected eyes (71.5%). Regarding severity, 62% presented in a mild form, while 9.9% were at a severe stage. About 88.2% presented with bilateralism.

**Conclusions:**

Most of the patients in their twenties presented with a mild bilateral form of the disease. This result is compatible with published international reports. It is recommended that the results of this study be considered when establishing a screening program in Palestine. Subsequently, patients will be identified at an appropriate time where action can be taken before disease progression take place.

## Background

Keratoconus (KC) has been traditionally classified as a non-inflammatory, degenerative ectatic condition of the cornea in which the cornea assumes an irregular conical shape. The visual consequences range from blurred vision to blindness if the condition is not treated [1]. In recent years, there has been an increasing amount of literature supporting the classification of KC as quasi-inflammatory (inflammatory-related) due to some biochemical changes [2–4]. It is likely to be a multifactorial, multigenic disorder with complex inheritance patterns, and environmental factors probably play an equally important role in disease causation [5–7]. Associations have been identified between KC and systemic conditions such as trisomy 21, Turner’s syndrome, cardiovascular diseases, various collagen vascular disorders and Marfan’s syndrome [6, 8].

Based on the literature as well as medical and ophthalmological expertise, it is noticed that KC is a major and common eye disease among the Palestinian population compared with other countries. This might be due to genetic and environmental risk factors, such as sun exposure (ultraviolet exposure) and nicotine [9]. Another factor associated with an increase in the risk of KC is consanguinity [10], which is prevalent in our country. Hence, the goal of this study is to determine the epidemiological characteristics of this disease in order to initiate a screening program based on well-organized data collected from our patients’ medical files.

## Methods

The medical files of 505 keratoconic patients were reviewed. The study participants were patients attending either the ophthalmology Centre of An-Najah National University Hospital (ANNUH) or the An-Noor Centre at the Specialized Arab hospital over the period from September 1, 2009 to January 1, 2016. They were presented as outpatients complaining of various visual symptoms. The diagnosis of KC was confirmed by the ophthalmologists depending on history, thorough slit lamp examination and, most importantly, Pentacam (Pentacam HR, Type 70,900, OCULUS, Germany). Subsequently, the appropriate management strategy was applied to each patient, ranging from spectacles and soft contact lenses to corneal transplantation, focusing on improving visual acuity to the greatest extent possible. Their epidemiological data at the time of diagnosis were collected and analysed, including residency, age at diagnosis, gender, best-corrected visual acuity (BCVA), severity and bilaterality of the disease. Disease severity was determined using the k median index (mean curvature power), which is widely used as a classification method in many studies [11], obtained from the Pentacam map. Thus, mild KC was defined as mean k < 48 diopters, moderate as 48–54 diopters and severe as > 54 diopters [12]. Regarding BCVA, eyes were classified into three main categories: the first one included eyes reported to have BCVA of ≤6/60 (20/200), the third category included those presenting with BCVA of ≥6/12 (20/40) and the second one in between [12]. According to BCVA and k readings, the data on the affected eyes only were included. Residency was categorized into city, village, and Palestinian refugee camps. It is worth mentioning that all keratoconic patients presenting to either centre in the targeted period were included in the study, so none were excluded. Before conducting the study, Institutional Review Board (IRB) approval was obtained from An-Najah National University ethics committee, and the required permission from both centres was obtained.

### Statistical analysis

Statistical analysis of the data was carried out using IBM SPSS V.20. Nominal variables were described using frequencies and percentages, while continuous variables were described by mean and standard deviation. Descriptive statistics, the chi-square test, independent-sample *t*-test, one-way ANOVA and Pearson’s correlation coefficient were used to analyse the data. Results were considered statistically significant at *P* ≤ 0.05.

## Results

The medical reports of 505 keratoconic patients, including 936 keratoconic eyes, included in our study were saved from 2009 to 2016; 61.6% of the patients presented in the last year, and the remaining 38.4% attended previously. This increase is explainable by the fact that ANNUH ophthalmic centre was established at the end of 2013.

### Residency

Depending on their residency, the patients were distributed among three groups (city, village and camp). The highest percentage, 67.9% (343/505), came from the city, while the lowest contribution, around 1.6% (8/505), was found to be from Palestinian refugee camps, and the remaining 30.1% (152/505) were villagers. As shown in Table 1, the residency for two patients was not recorded. A significant difference in k readings was detected between patients from cities, villages and Palestinian refugee camps (*p* = 0.041), reflecting that patients from villages were reported to have higher mean k readings than patients from cities and Palestinian refugee camps.

**Table 1:** Geographical distribution of the sample (*n* = 505)

	Frequency	Percentage (%)
Residency		
City	343	67.9
Village	152	30.1
Palestinian refugee camps	8	1.6
Missing	2	0.4
Total	505	100

### Distribution by age group at the time of diagnosis

**Fig. 1:**
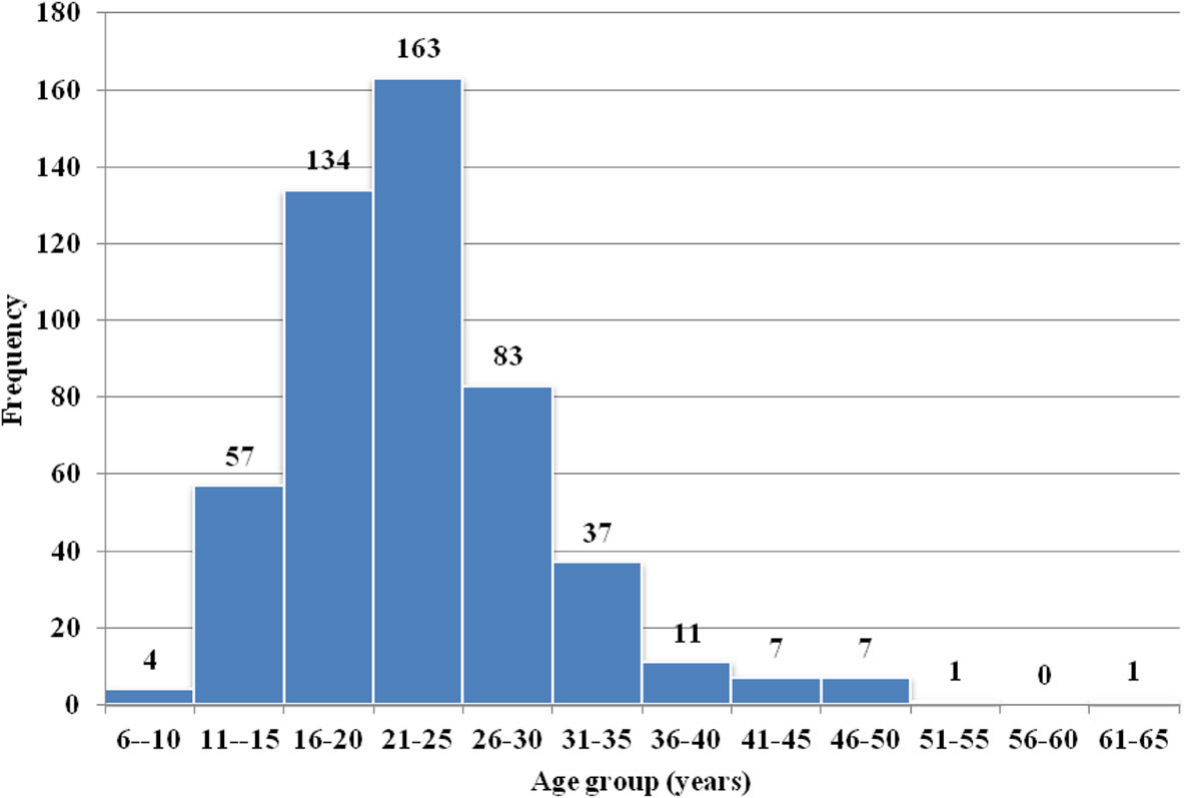
Sample distribution among age groups at the time of diagnosis (*n* = 505)

The age range variable was divided into a set of categories, each 5 years in length. The age at presentation ranged from 8 to 62 years, with a mean (SD) age of 23.33 (7.37) years. It is worth mentioning that around 75% of them presented between 16 and 30 years of age with high prevalence rate among this age group (21–25), to which around 32.3% of patients belonged, followed by the age groups 16–20 and 26–30, with 26.5% and 16.4%, respectively. A dramatic decrease was noticed at both age margins, especially when patients aged (Fig. 1).

### Sex distribution, BCVA, severity and bilaterality of KC at the time of diagnosis

There was a nearly equal distribution of patients between the two sexes (49.5% males, 50.5% females). On initial evaluation, the BCVA was recorded as ≥6/12 in the majority of affected eyes, around 71.5% (670/936). On the other hand, only 2.4% (21/936) presented with ≤6/60 BCVA, and the rest 16.5% (155/936) were determined to have > 6/60 to < 6/12 BCVA. BCVA data were missing in 9.6% (90/936) of cases. Regarding severity, most of the affected eyes, 62% (580/936), presented with the mild form, while only 9.9% (93/936) had the severe form and an intermediate percentage, 28.1% (263/936) had the moderate form. About 88.2% presented with bilateralism.

### Demographic characteristic of patients in relation to sex

Generally, it is worth mentioning that the only significant difference in the epidemiological parameters (age at presentation, severity and bilaterality of KC, BCVA) between the two sexes was detected in severity (*P* = 0.030), with female being more vulnerable to presenting with severe and moderate forms. Otherwise, no significant differences in the rest of parameters were found between the two sexes. For both sexes, the highest contribution was that of the mild form and was higher among males than females (66% vs 58.1%). On the other hand, the lowest contribution was that of the severe form for both sexes and was higher among females than among males (10.7% vs 9.20%). A closer examination of each parameter in turn revealed that the mean (SD) ages of the male and female groups were 22.98 (7.53) and 23.37 (7.15) years, respectively. Around 70% of patients presented at an age of ≥20 years between both sexes. According to BCVA at the time of diagnosis, in both sexes most diseased eyes detected had BCVA ≥6/12, around 72.1% of male eyes compared with 71.1% of female eyes. Meanwhile, the lowest contribution in each sex was by those with BCVA ≤6/60: around 2.6% of males and 1.9% of females. Regarding bilateralism, around 88.4% of males and 88.1% of females were found to have KC in both eyes (Table 2).

**Table 2:** Demographic characteristics of the study population in relation to sex

	Total *n* = 505 (%)	Male *n* = 250 (%)	Female *n* = 255 (%)	*P* value^a^
Variable				
Mean age (year) ± SD	23.33 ± 7.37	22.98 ± 7.53	23.37 ± 7.15	0.548^e^
Age at presentation (year)^b^				
< 20	151(29.9)	79(31.6)	72 (28.2)	0.408^f^
≥20	345 (70.1)	171(68.4)	183(71.8)	
Bilateralism				
Yes	443(88.2)	221 (88.4)	222(88.1)	0.916^f^
No	62 (11.8)	29 (11.6)	33(11.9)	
Severity of KC at presentation^c^				
Severe (k > 54)	93 (9.9)	42(9.2)	51(10.7)	0.030^f^
Moderate (k = 48–54)	263 (28.1)	114(24.3)	149 (31.2)	
Mild (k < 48 D)	580 (62)	303 (66)	277 (58.1)	
BCVA^c, d^				
≤6/60	21(2.4)	12 (2.6)	9 (1.9)	0.975^f^
> 6/60 to < 6/12	155 (16.5)	73(15.9)	82 (17.3)	
≥6/12	670(71.5)	331(72.1)	339(71.1)	

*Abbreviations*: *SD* standard deviation, *KC* Keratoconus, *BCVA* best-corrected visual acuity

^a^The *P*-value is bold where it is less than the significance level cut-off of 0.05

^b^20 years is used as the cut-off point as in most of the studies [12]

^c^Values of non-diseased eyes were not entered

^d^Missing data for 90 patients

^e^Statistical significance of differences calculated using the independent-samples *t*-test

^f^Statistical significance of differences calculated using the Pearson’s chi-squared test

### Correlation between different demographic parameters

Application of the chi-square test indicated that there was no statistically significant association between sex and the BCVA (*P* = 0.665, χ^2^ = 0.815). Using Pearson’s correlation, a statistically significant mild correlation was found between K average readings (severity of KC) and age (*P* = 0.001, *r* = − 0.108), in which patients who presented at earlier ages had a more severe form of KC than did older patients. Furthermore, a statistically significant moderate negative correlation was found between severity of KC and the BCVA (*P* = 0.004, *r* = − 0.602); thus, as the severity of the disease increased, the BCVA that could be achieved by the patient decreased.

## Discussion

Keratoconus is a serious and common eye disease among the Palestinian population compared with those of other countries; this may be due to genetic and environmental risk factors, such as sun exposure (ultraviolet exposure), as well as consanguinity [8]. It usually manifests during late adolescence or the early twenties, with a gradually slowing progression for 10–20 years from diagnosis [13]. It is a painless disease that, in cases of late diagnosis and management, may end with severe visual loss due to the associated high short-sightedness and irregular astigmatism [14]. Therefore, it is worthwhile to highlight this issue by studying the epidemiological characteristics of all keratoconic patients attending two major ophthalmology centres (at ANNUH and an-Noor centre at the Specialized Arab hospital) in the West Bank, Palestine over the period from September 1, 2009 to January 1, 2016. This, in turn, may help in establishing a screening program for this widespread and serious disease, based on well-organized data collected from these main ophthalmic centres in our country. Subsequently, patients will be identified at an appropriate time when action can be taken before disease progression takes place.

This study found a mean age at the time of diagnosis of around 23.33 ± 7.37 years, and no significant difference was found in the mean age between sexes (*P* = 0.548). Our result was nearly consistent with a Malaysian study, where the mean age of disease onset was 20.9 ± 5.6 years [15]. Meanwhile, in Saudi Arabia, the mean age at diagnosis was slightly lower than in our study, about 17.7 years for males and 19.0 years for females [16]. On the other hand, a higher mean age, around 27 years, was detected in Caucasian populations, suggesting a later disease onset [16], which is also seen in Macedonia, as the mean age at the time of disease detection was 26.81 ± 1.25 [17]. In general, our results agreed with those of internationally published studies concerning the early age of onset [18]. Several possible reasons could be cited here to explain these differences, including genetics and environmental and geographical factors, such as consanguineous marriages and ultraviolet exposure, which are considered the main risk factors for KC development and thus may explain the much earlier age of onset in the Saudi Arabian study compared with other studies.

Most of the patients in this study, as well as those in the Saudi Arabian [16], Malaysian [15] and Macedonian [17] studies presented with the mild disease form at the time of diagnosis, for example, in this study, 62% had the mild form, 28.1% had moderate KC and 9.9% of the total patients’ eyes had severe KC. Moreover, in Malaysia 37.6%, were stage I, 30.1% stage II, 4.4% stage III and 27.8% stage IV at the time of diagnosis [15]. Also, in Saudi Arabia it was found that 39.2% were in the early stage, 42.5% in the moderate stage and 18.3% in the advanced one [16]. Finally, In Macedonia 52.08% were in the mild stage, 36.45% were in the moderate stage and 11.57% were in the late stage [17]. In general, most of the patients in the four studies had a mild to moderate form of the disease at the time of diagnosis, with some variability. This finding can be explained by several factors: firstly, this might be due to ophthalmologists’ awareness concerning KC, as they have a low threshold for investigation to capture early-stage patients. Secondly, it could be due to an accidental discovery during a routine check-up of visual acuity or when changing glasses, since most KC patients are already myopic and must change their glasses frequently due to changes in refractive error. Finally, the increasing trend towards refractive surgeries in myopic patients raises the likelihood of KC discovery, as preoperative assessment by Pentacam is mandatory for all patients before these surgeries.

As detected in this study, there is a significant mild negative correlation between the mean k readings and the age at which the patient presented with KC (*P* = 0.001, *r* = − 0.108); therefore, the younger age group is more likely to develop a severe disease stage than the older one. Our result was consistent with those found in the Saudi Arabian study [16] and Collaborative Longitudinal Evaluation of Keratoconus (CLEK) Study [19]. As stated in the CLEK study, age is considered a key factor in severity-related outcomes in KC, as persons diagnosed with KC at a younger age are more vulnerable to needing penetrating keratoplasty in a shorter time compared with older patients, as disease progression will be faster [19]. Thus having a disease in the early years of life means entering a very rapid progression to an early very severe form of the disease; meanwhile, a gradual regression in severity was noticed as the patients aged, which justifies that developing a disease at a later age offers a greater possibility of a very slow disease progression and subsequently remaining in the mild to moderate stages and less likely to progress to the severe form. In general, our results were consistent with the fact that KC progresses slowly and then gradually stops in the 10–20 years following diagnosis [13].

Our results indicated significant differences between male and female groups regarding the severity of KC (*P* = 0.030). Thus, male patients are more likely to present with mild disease than females. Sex-based differences regarding KC might be due to hormonal changes during pregnancy, which induce KC progression. In addition, females tend to delay visiting the physician, frequently complaining of being busy all the time; thus, males will be identified more often in the milder form than females. Additionally, resistance to wearing glasses, as they are considered to be a social stigma for them, and prevents them from visiting ophthalmology clinics until the late stage.

Most of the eyes in this study (71.2%) had BCVA of ≥6/12; this result was similar to those of the Saudi Arabian study, in which 100% of the eyes achieved a corrected VA 6/12 or better, and 33% of eyes achieved 6/6 or better with glasses [16]. As well, BCVA was 6/9 in the Malaysian study [15]. This study also found a significant negative correlation between severity of KC and the best spectacle visual acuity achieved (*P* < 0.005, *r* = − 0.602). The clear majority of the patients in these three studies have a better chance of correcting their visual acuity at the time of diagnosis. This may be due to the above-mentioned results, as most of these study patients have a mild form of the disease at the onset of diagnosis and subsequently less vision deterioration and a better chance of achieving vision correction. In fact, KC progression negatively affects many important aspects of life besides visual deterioration, such as educational and employment opportunities.

Many keratoconic cases start unilaterally but eventually affect the contralateral eye [20], although this may occur years after the initial disease detection [14]. The prevalence of unilateral KC ranges from 14.4% to 41% [12]. This was apparent in our results, as bilateral disease was found in 87.7% of patients, with no significant association between gender and bilaterality of the disease (*P* = 0.916). This was consistent with other studies, as the bilateral appearance of KC at the moment of diagnosis was present in 84.4% of patients in the Macedonia study [17], and around 77% of cases were bilateral in the Malaysia study [15, 16]. Having these high percentages of bilaterality in these three studies at the time of diagnosis might be a result of missdiagnosis of the disease in the very early stages; thus, it was discovered late enogh to be developed in the fellow undiseased eye and present as being bilateral at the time of diagnosis. Whatever the cause, the end result justifies the importance of examination and Pentacam assissment of both eyes with regular follow-up to avoid missing the disease in the fellow undiseased eye.

Regarding residency, a significant difference in k readings between residents of cities and villages were found, with villagers having a higher probability of presenting at a late stage of KC; this result should prompt us to give more attention to villagers regarding awareness and implementation of a screening program.

### Strengths and limitations

To the best of our knowledge, this is the first study to describe the characteristics of keratoconic patients in Palestine. Even though the main objective of our study, which was to studying the epidemiological characteristics of keratoconic patients, was achieved, as any study, it has certain points of limitation. The first one concerns generalizability, since the data were only drawn from two centres; however, we believe this was not a serious concern because these centres are the major ones in our country and receive most of the patients. Secondly, missing data can occur with any retrospective study. However only a few epidemiological and demographic characteristics—sex, bilaterality and residency—were presented and evaluated in the study. Including other epidemiological factors, such as family history, consanguinity, associated systemic diseases, history of atopic disease and eye rubbing, would have provided more valuable information and is recommended for future studies.

## Conclusions

Most of the patients presented with a bilateral mild stage of the disease in their second decade. Male patients are more likely to present with a mild stage of the disease than females. In addition, the younger age group has a higher probability of progressing rapidly to the severe stage. These results require our urgent attention to employ a well-organized, accessible and inexpensive screening program at early ages, for example, at school and university and, most importantly, to increase public awareness regarding this issue by several methods, including conducting lectures and printing brochures, among vulnerable people.

## Abbreviations

ANNUH: An-Najah National University Hospital
BCVA: best-corrected visual acuity
IRB: Institutional Review Board
KC: Keratoconus
